# Monocytic Cells Become Less Compressible but More Deformable upon Activation

**DOI:** 10.1371/journal.pone.0092814

**Published:** 2014-03-27

**Authors:** Agnese Ravetto, Hans M. Wyss, Patrick D. Anderson, Jaap M. J. den Toonder, Carlijn V. C. Bouten

**Affiliations:** 1 Department of Biomedical Engineering, Eindhoven University of Technology, Eindhoven, the Netherlands; 2 Department of Mechanical Engineering, Eindhoven University of Technology, Eindhoven, the Netherlands; 3 Institute for Complex Molecular Systems, Eindhoven University of Technology, Eindhoven, the Netherlands; Dalhousie University, Canada

## Abstract

**Aims:**

Monocytes play a significant role in the development of atherosclerosis. During the process of inflammation, circulating monocytes become activated in the blood stream. The consequent interactions of the activated monocytes with the blood flow and endothelial cells result in reorganization of cytoskeletal proteins, in particular of the microfilament structure, and concomitant changes in cell shape and mechanical behavior. Here we investigate the full elastic behavior of activated monocytes in relation to their cytoskeletal structure to obtain a better understanding of cell behavior during the progression of inflammatory diseases such as atherosclerosis.

**Methods and Results:**

The recently developed Capillary Micromechanics technique, based on exposing a cell to a pressure difference in a tapered glass microcapillary, was used to measure the deformation of activated and non-activated monocytic cells. Monitoring the elastic response of individual cells up to large deformations allowed us to obtain both the compressive and the shear modulus of a cell from a single experiment. Activation by inflammatory chemokines affected the cytoskeletal organization and increased the elastic compressive modulus of monocytes with 73–340%, while their resistance to shape deformation decreased, as indicated by a 25–88% drop in the cell’s shear modulus. This decrease in deformability is particularly pronounced at high strains, such as those that occur during diapedesis through the vascular wall.

**Conclusion:**

Overall, monocytic cells become less compressible but more deformable upon activation. This change in mechanical response under different modes of deformation could be important in understanding the interplay between the mechanics and function of these cells. In addition, our data are of direct relevance for computational modeling and analysis of the distinct monocytic behavior in the circulation and the extravascular space. Lastly, an understanding of the changes of monocyte mechanical properties will be important in the development of diagnostic tools and therapies concentrating on circulating cells.

## Introduction

Atherosclerosis is a chronic inflammatory disease that affects mainly large and medium sized arteries. Circulating monocytes, which form a small subpopulation of the leukocytes, are well known to be involved in the disease progression [1], [2]. The rheological properties of monocytes play a significant role in flow dynamics and alterations in cell mechanical properties, such as cell deformability, can significantly influence vascular flow and might lead to vascular complications, such as sequestration of monocytes on the vessel wall.

Investigating the evolution of monocyte mechanical behavior might therefore lead to a better understanding of the progression of the disease and the development of viscoelastic mechanical models clarifying the abnormal behavior of monocytes in circulation. During the process of inflammation, monocytes become activated in the blood stream. Upon activation, they adhere to the endothelium and, in a process called diapedesis, extravasate through the endothelium to migrate to the target tissue. The interaction of monocytes with the blood flow and with endothelial cells results in structural changes in the cytoskeleton. In fact, the cytoskeleton has the ability to rapidly regulate the amount and the architecture of its protein components in response to inflammatory cytokines to enable cells to fulfill their function [3].

For the main leukocyte subpopulations, neutrophils and granulocytes, it has been established that reorganization of cytoskeletal proteins, in particular of the microfilament structure, is strongly related to changes in cell shape and mechanical properties [4]–[11]. We hypothesize that a similar response is present in the monocytic subpopulation.

Since most of the studies on leukocytes focus on neutrophils or granulocytes, there is little knowledge on the relation between the activation and the mechanical properties of monocytes [5], [12], [13]. Doherty *et al.* measured the stiffness of monocytes upon activation using a cell poker. They showed that monocytes become stiffer upon stimulation with lipolysaccharide, which results in increased monocyte retention in the capillaries. On the other hand, Rinker *et al.* showed that linoleic acid, known for its pro-inflammatory effect, increases monocytic cell deformability as measured by micropipette aspiration; such increased deformability may promote adherence, as it increases the surface area available for bond formation. None of these studies evaluated the full elastic response of the cells, instead focusing on a single mode of cellular deformation. Thus, in order to elucidate and understand the changes in circulating cell mechanical properties that occur upon activation, it is of key importance to experimentally access the full elastic response of the cells, thereby characterizing the response to both compressive and shear deformations.

In this study, the changes in the mechanics of monocytes upon cell activation were studied systematically, characterizing the full elastic response of the cells. Structural changes accompanying these changes in mechanics were visualized by imaging the re-organization of actin in the cells. For the mechanical measurements a technique termed Capillary Micromechanics [14]–[16] was used, which measures the pressure-induced deformation of cells as they are forced through a tapered glass microcapillary. This recently developed technique has the advantage of obtaining data at the single cell level and characterizing both the compressive and the shear moduli from a single experiment. Further, the approach allows evaluation of cell mechanical properties over a large range of deformations, spanning the entire physiologically relevant range of deformations.

Validation experiments using monocytes with a disrupted actin cytoskeleton indicated that the technique was valid to determine changes in cellular elastic behavior upon cytoskeletal structural changes. In addition, the application of inflammatory cytokines caused rapid changes in actin amount and organization and, consequently, changes in mechanical behavior. It was found that the compressive modulus was higher for activated cells, but their resistance to shape deformation, characterized by the elastic shear modulus, was lower. This effect was especially pronounced at high strains, such as those occurring during diapedesis through the vascular wall. These results could explain previous seemingly contrasting results on circulating cell mechanical behavior in literature, which could not distinguish between deformability and compressibility of the cells.

Since our experiments provide a link between cell mechanical properties and behavior, they are of special interest for the development of quantitative models that describe and predict the behavior of monocytes in the circulation and they might lead to a better understanding of the role of circulating monocytes in the progression of inflammatory diseases.

## Materials and Methods

### Study design

The effect of monocyte activation was assessed using Capillary Micromechanics and correlated to the actin cytoskeletal organization in cultured cells. First, the validity of the capillary micromechanics technique was assessed by investigating the difference in mechanical properties between non-treated monocytic cells (**NT**, cell number = 7) and cytochalasin-D-treated monocytic cells (**cytoD**, cell number = 3). Cytochalasin-D is a drug known to disrupt actin filaments and, consequently, to influence cell mechanical properties.

After validation, the influence of activation with inflammatory cytokines on cell mechanical properties was assessed by investigating differences in compressive and shear moduli between non-treated monocytic cells (**NT**, cell number = 7) and cells treated with Lipopolysaccharides (**LPS**, cell number = 5).

In analogy with previous studies in literature, both the validation and the activation experiment were performed at room temperature (21°C). As it is recognized that cell mechanical properties may change with temperature, the experiments were repeated at 37°C to mimic physiological temperature conditions. Analysis of NT (cell number = 5), LPS (cell number = 5) and CytoD (cell number = 2) treated cells at 37°C indicated that this temperature increased the sensitivity to activation. Now, even non-treated control cells became activated due to mechanical deformation, thereby hampering the comparison with LPS-induced activation on cell mechanical properties. NT cells formed pseudopods upon deformation (Fig. S1) and demonstrated unusual mechanical behavior due to late activation in the capillary tip, especially at large strains (Fig. S2 and Fig. S3). For this reason, comparisons between NT, LPS and CytoD treated cells in the Results section are provided for the experiment performed at room temperature. Interestingly, however, analysis of LPS-treated cells at 37°C indicated that the increase of experimental temperature resulted in decreased resistance to deformation upon activation (Fig. S4).

### Cell culture and treatment

Human promyelocytic leukemia HL60 cells were used as a model cell line for monocytes. The cells were obtained from the European Collection of Cell Cultures (ECACC) and cultured in RPMI 1640 medium (Invitrogen, Landsmeer, Netherlands) with 2 mM L-glutamine (Lonza, Verviers, Belgium) and 10% Fetal Bovine Serum (Greiner, Alphen a/d Rijn, Netherlands). Cell cultures were maintained at concentration between 1–9×10^5^ cells/ml at 37°C in a humidified atmosphere containing 5% CO_2_. Cell differentiation towards monocytic lineage was stimulated by treatment with 0,4 mM Sodium Butyrate (Sigma, Zwijndrecht, Netherlands), as previously described [17], [18], for 4 days at culturing conditions prior to validation and activation experiments.

Cell actin cytoskeleton was disrupted by exposure to 4 μM Cytochalasin-D (Cyto-D, Sigma, Zwijndrecht, Netherlands) for 20 minutes at 37°C. Cell activation was induced by incubation with 2 μg/ml Lipopolysaccharides (LPS, Sigma, Zwijndrecht, Netherlands) for 15 minutes at 37°C.

### Measurement of filamentous and monomeric actin

To characterize cytoskeletal organization, cells were centrifuged at 150 *g* for 5 minutes, resuspended in 4% paraformaldehyde and incubated for 10 minutes at room temperature. The cells were permeabilized for 15 minutes with 0.1% Saponin (Sigma, Zwijndrecht, Netherlands) in PBS. Next, cells were blocked for 20 minutes with 3% bovine serum albumin (BSA) and incubated for 30 minutes with Alexa-555-labeled Dnase I (10 μg/ml, Sigma, Zwijndrecht, Netherlands) and Alexa-488-labeled Phalloidin (50 μg/ml, Sigma, Zwijndrecht, Netherlands) for staining of globular and filamentous actin, respectively. A quantitative measure of fluorescence was obtained using FACS flow cytometry (Millipore, Guava EasyCyte HT), while fluorescent microscopy was used to imaging the cytoskeletal structure (Zeiss Axiovert 200 M).

### Single cell cytology

The cytoskeletal structure of the cell during cell deformation was visualized with actin staining while the cell was compressed at the tip of the capillary. Cells were stained at two different pressures to evaluate protein remodeling at increasing pressure and to observe the effect of treatment on the cytoskeleton.

First, a cold solution of 4% paraformaldehyde was placed near the capillary outlet to fix the cell by diffusion through the tip. After 10 minutes incubation, cell permeabilization was performed by incubation for 10 minutes with a solution of 0.5% Triton-X 100 (Merck, Amsterdam, Netherlands). The droplet at the tip was then replaced by a staining solution of Alexa-488-labeled Phalloidin (50 μg/ml, Sigma, Zwijndrecht, Netherlands) for actin, and DAPI (Sigma, Zwijndrecht, Netherlands) for staining the nuclei. The organization of cytoskeletal protein and the conformation of the cell nucleus were then evaluated by fluorescence microscopy (Zeiss Axiovert 200 M).

### Capillary micromechanics

#### 1. Microcapillary device

**Figure 1:**
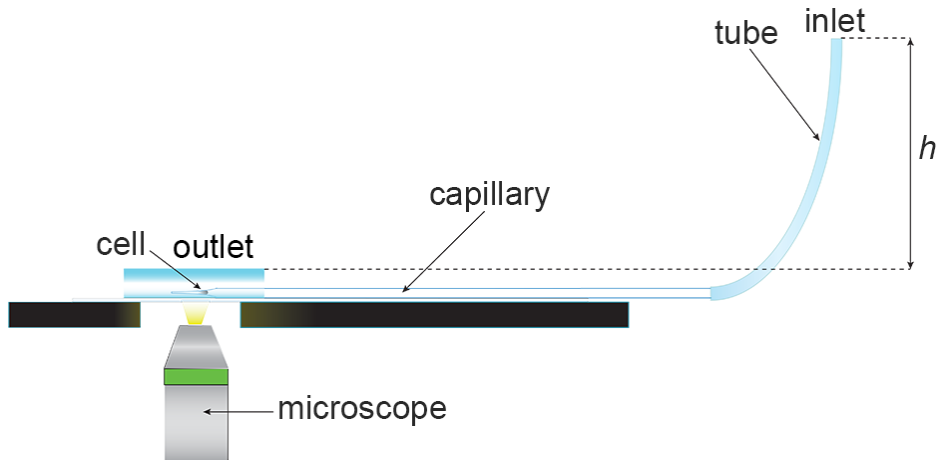
Capillary micromechanics setup. Capillary micromechanics; schematic of experimental setup. In brief: The tip of a horizontally stabilized tapered capillary (3-μm-wide) leads into a liquid drop of buffer solution (PBS). A cell is lodged in the tapered part of the capillary. A flexible tube is attached at the backside of the capillary. The filling height h of PBS buffer in the tubing determines the pressure difference in the system (1 mm H2O is 10 Pa, changed in steps of 10 Pa) and thereby the externally applied stress acting on the cell. Cells are imaged in real-time (shape and volume) using an inverted microscope (40x objective) at each pressure step after a cell lodges in the capillary.

A device based on cell deformation in a tapered microcapillary was used to measure the mechanical properties of circulating cells [14], [15]. A schematic of the setup is shown in Fig. 1. The device consists of a borosilicate micropipette tapered to a 3 µm tip. The inlet of the microcapillary is connected to a flexible tube by which the pressure difference between the inlet and the outlet of the capillary is adjusted, as shown schematically in Fig. 1. The cell suspension is then flown into the device by applying a small overpressure. As the cells are larger than the tip of the capillary, the first cell arriving near the tip becomes lodged in the tapered capillary. By adjusting the fluid height *h* in the tube, the pressure difference and, consequently, the externally applied stress on the cell can be regulated. For each experiment, the pressure was increased in small steps from initial pressure of 10 Pa to the final maximum pressure of 500 Pa and the cell was allowed to equilibrate for 120 seconds between steps. Cell deformation was then assessed at the end of the stabilization period by microscopy imaging (Zeiss Axio Observer Z1) followed by a digital image analysis, yielding the volumetric and shape deformation of the cells in terms of the cell volume *V*, as well as the radial and longitudinal strain deformations *ε_r_* and *ε_z_*, respectively.

#### 2. Capillary micromechanics and friction analysis

In equilibrium, the externally applied stresses induced by the fluid pressure difference *p* must balance the internal stresses in the material. In the absence of friction, these internal stresses are readily expressed as a function of the elastic stresses and strain deformations. The pressure *p_wall_* exerted by the cell on the glass walls of the capillary is obtained by balancing the direct external force *F_p_  =  pπR^2^*, induced by the applied pressure difference *p*, with the longitudinal component of all contact forces between the cell and the surface of the glass capillary, approximated as *F_//_ ≈ 2πRLsin(α)p_wall_* [14]. With *R* the (average) radius and *L* the length of the contact area between the cell and the glass wall, and *α* the tapering angle of the capillary, this balance yields a wall pressure of *p_wall_ ≈ R_p_ /2Lsin(α)*. As a result, both the compressive (*K*) and the shear (*G*) elastic moduli can be calculated directly from the cell deformations and the stresses that are induced as the applied pressure increases. The compressive modulus reflects the resistance of the cell to a volume change, while the shear modulus represents the resistance to a shape change. The compressive and shear moduli can then be expressed as 
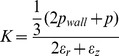


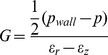



where *p* is the fluid pressure, *p_wall_* is the pressure that the wall of the microcapillary exerts on the cell and *ε_r_* or *ε*_z_ are the strains in radial and longitudinal direction, respectively [14].

**Figure 2:**
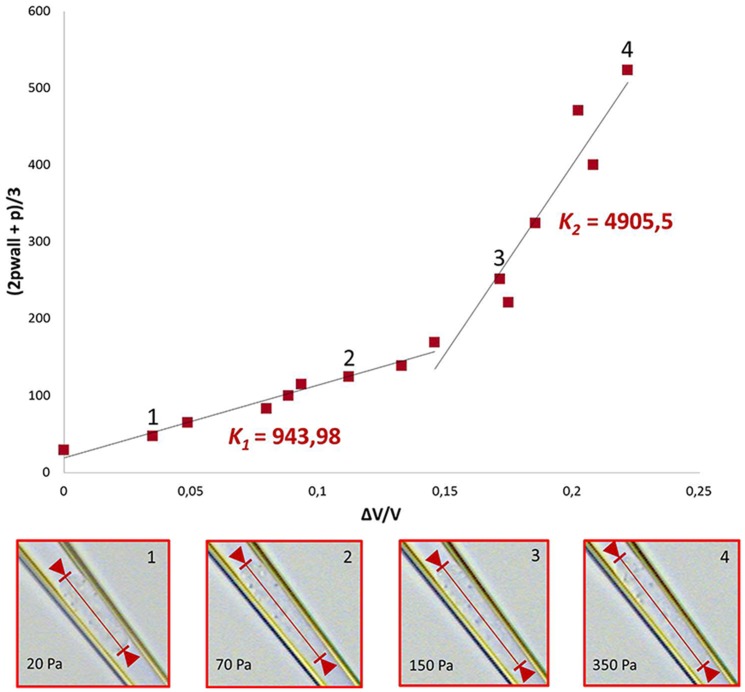
Calculation of modulus and deformation under pressure. Since the mechanical properties of cells depend on the degree of deformation, the curves representing the compressive and the shear moduli were non-linear. For this reason, the moduli were calculated using two different linear fits in two different ranges of the deformation, shown in the top graph for the case of non-treated cells. These fits yield two significantly different values for the compressive modulus K (K1 and K2). The bottom four graphs display representative images showing cell deformation of a not-treated cell at room temperature, as the pressure increases (20, 70, 150, 350 Pa). The corresponding locations in the stress-strain are indicated as numbers in the main top graph.

The mechanical properties of cells often depend on the degree of deformation, displaying strain-hardening behavior at larger deformations. To study this effect in response to activation, the moduli *K* and *G* were calculated using two different linear curve fits per cell: one for smaller deformations (*K*_1_, *G_1_*) and one of the linear region of the curve at larger deformations (*K*_2_, *G_2_*), respectively, as exemplified for *K* in Fig. 2. This figure also shows images of a deformed cell as pressure increases.

**Figure 3:**
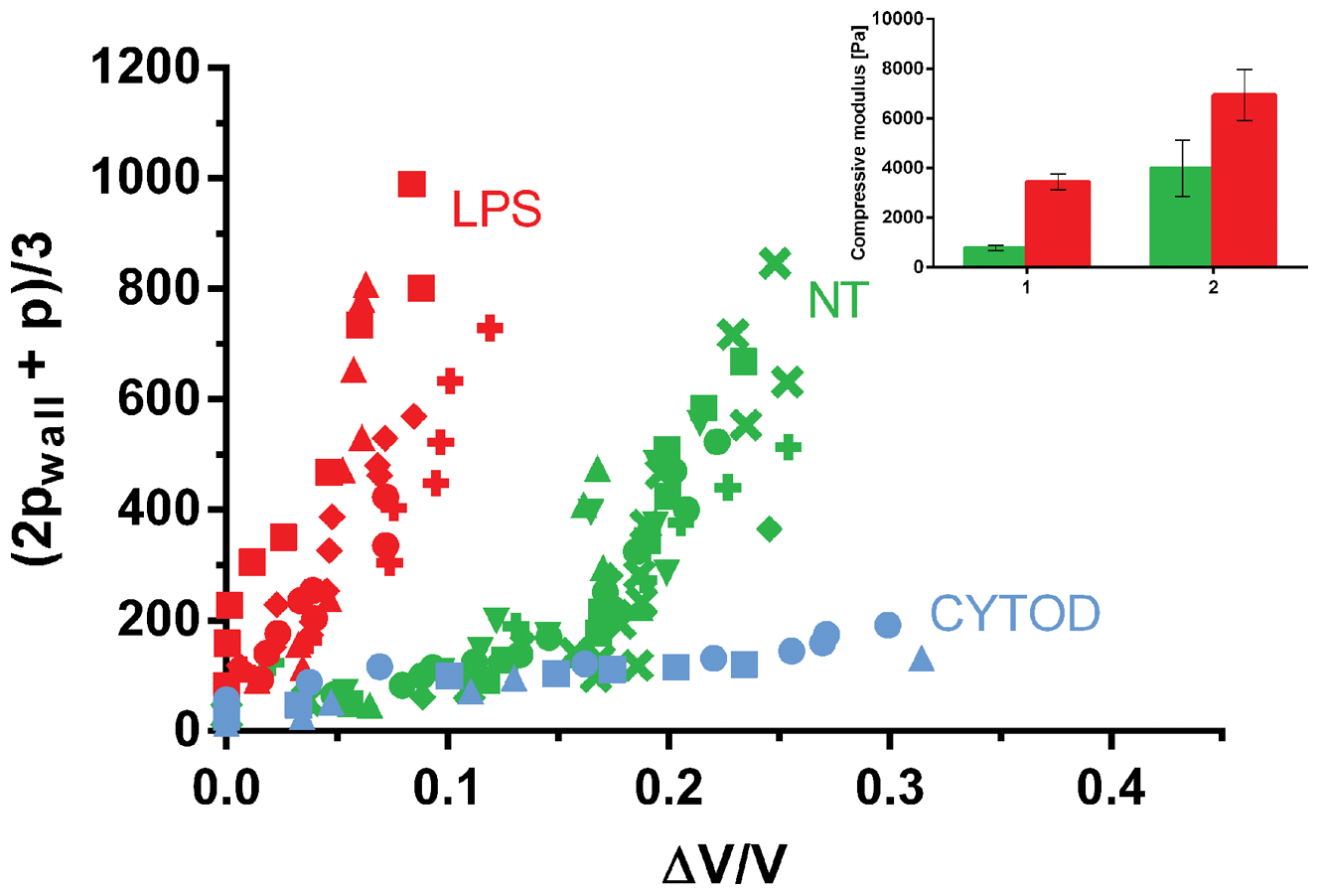
Compressive modulus. Scatterplot of the compressive stress versus the volumetric strain for non-treated (NT, GREEN), activated LPS-treated (RED) and cyto-D treated (BLUE) cells. To quantify the effect of activation, the compressive moduli K1 and K2 for the NT (cell n = 7) and LPS-treated cells (cell n = 5) are shown (insert). The compressive moduli were calculated by two linear fits to the data, as illustrated in Fig.2.

**Figure 4:**
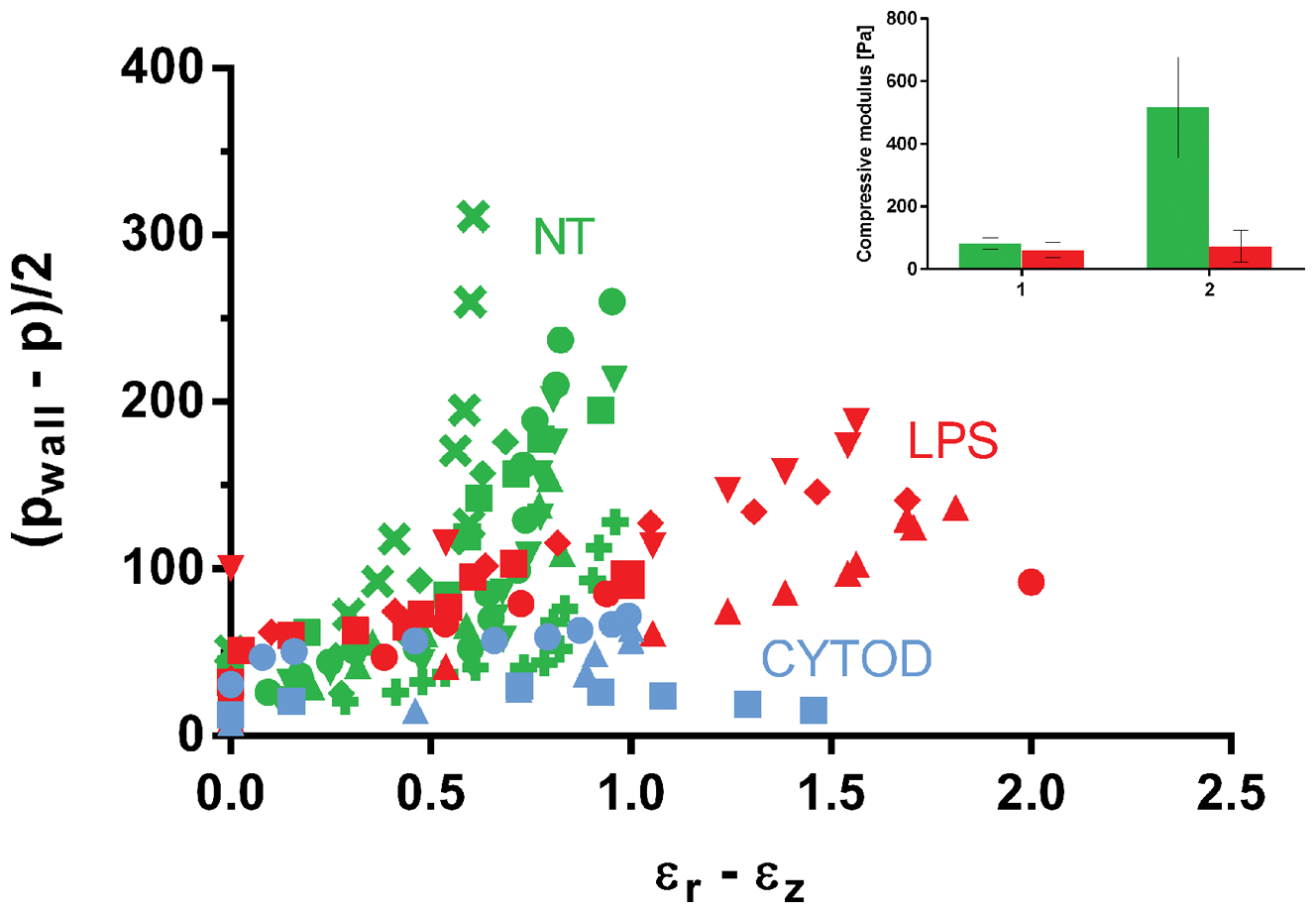
Shear modulus. Scatterplot of the characteristic differential stress (p_wall_ – p)/2 as a function of strain ε_r_ – ε_z_ for not-treated (GREEN), activated LPS-treated (RED) and cytoD-treated (BLUE) cells. In the insert of the figure, the quantification of the shear modulus for not-treated (cell n = 7) and activated cells (cell n = 5) is shown. The shear modulus was calculated by two linear fits, as shown in Fig.2.

Data are presented as stress-strain scatter plots for each applied pressure (Figs. 3–4). The compressive and shear moduli then correspond respectively to the slopes of the characteristic stress as a function of the characteristic strain for each of the modes of deformation probed. Specifically, the compressive modulus K is evaluated by plotting the characteristic stress defined by *(2p_wall_ + p)/3* as a function of the deformation *ΔV/V*, as shown in Fig. 3. The compressive modulus then corresponds to the slope of this curve. In analogy, to extract the shear modulus, the characteristic stress *(p_wall_ – p)/2* is plotted as a function of the strain *(ε_r_ – ε_z_)*, as shown in Fig. 4, where *G* is given as the slope of the curve. The values of the linear fitting are presented as mean ± SD for the linear regions of the curve (Figs. 3–4, insert).

The capillary and the tubing were coated with 2% BSA to minimize friction and adhesion of cell to the wall of the capillary. Moreover, to verify the absence of friction, cells were flown in a flat non-tapered 4 μm-wide glass capillary to observe the critical force that causes them to move and to check whether friction could have an influence on the calculation of the elastic moduli. It was found that the minimum applied pressure used in the experimental method (10 Pa) was enough to set the cell in motion and that the cell kept moving in the capillary (See Video S1). As a result, friction between the cell and the glass capillary was neglected in the calculation of the elastic moduli.

## Results

### Actin polymerization and organization depend on cell activation

Actin organization was evaluated in order to investigate the effect of structural change on the elastic response. For the validation study, HL60 cells were treated with 4 μM Cyto-D to disrupt the actin structure.

**Figure 5:**
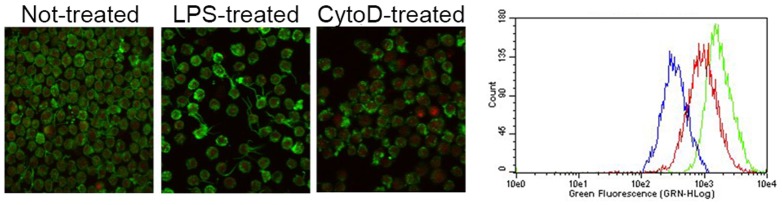
Actin analysis: immunohistochemistry and FACS. A) Representative immunofluorescent images of HL60 cells showing structural organization of F-actin (green) and G-actin (red). B) Typical intensity distribution of F-actin for NT cells (red), LPS-treated cells (green) and CytoD-treated cells (blue) as measured with flow cytometry.

As shown in Fig. 5A, untreated monocytic cells display a diffuse shell of filamentous F-actin structure, while G-actin is distributed throughout the cytoplasm. After treatment with Cyto-D, a clear loss of actin structure and organization is observed. After treatment with LPS, an increase in actin polymerization and a structural reorganization of F-actin localized at the cell cortex could be observed. Some cells exhibit projection of pseudopods, namely actin-rich structures, which are characteristic of the activated state of monocytes.

These results were confirmed by FACS analysis, as shown in the typical fluorescence intensity distributions in Fig. 5B. While the Cyto-D-treated group shows a clear decrease of F-actin, the LPS-treated group exhibits a pronounced increase in F-actin, compared to the untreated group.

**Figure 6:**
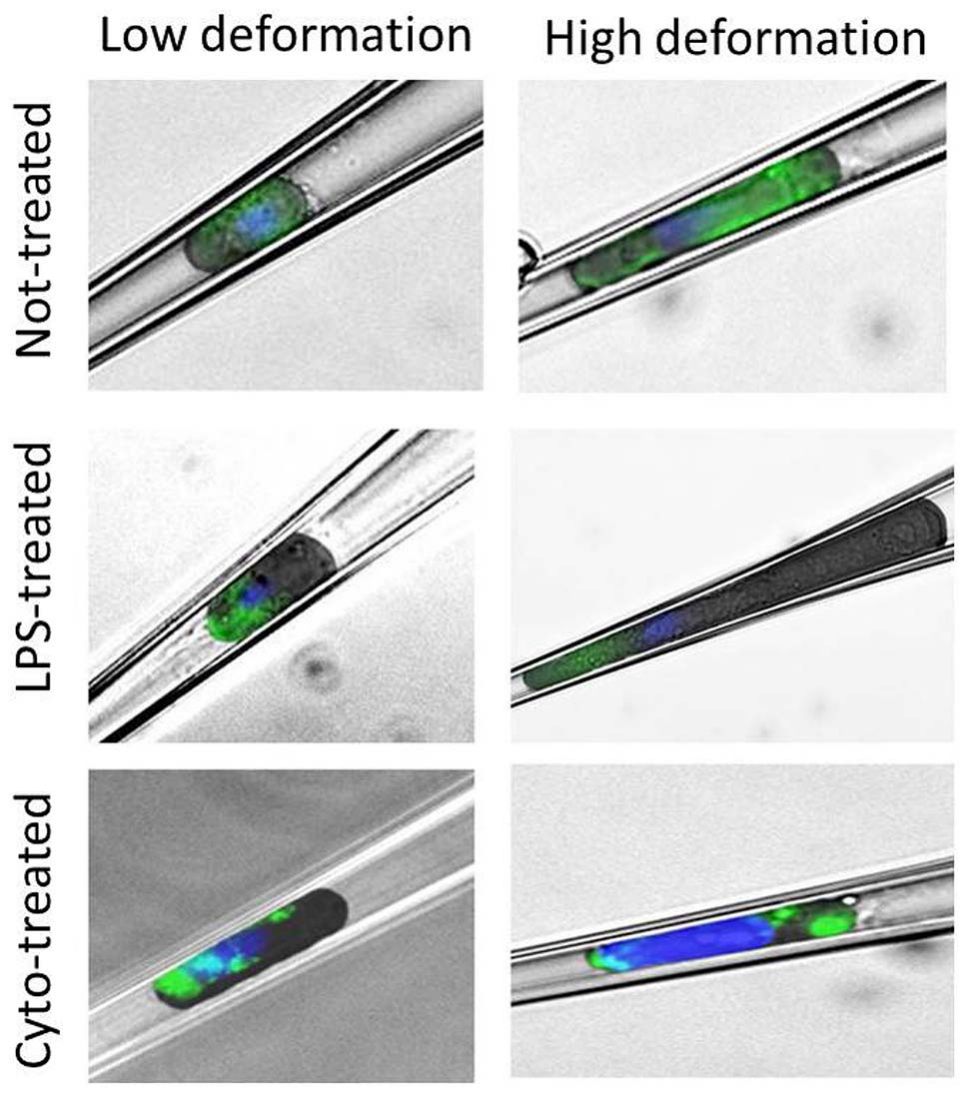
Actin staining in pipette. Immunostaining of cells deformed in the micropipette. Deformation at 20 Pa (left) and at 350 Pa (right) are shown for each cell group. The images were obtained by overlay of a bright field image and a fluorescent image. Actin structure is stained in green. Nuclei are stained in blue.

Mechanical deformation itself might also have a strong effect on cytoskeletal remodeling and disruption of cytoskeletal filaments. For this reason, the cells were also stained while being compressed in the tip of the capillary. The results showed that actin is uniformly distributed in non-treated cells, while LPS induces polarization of actin structure to the front of the cell (Fig. 6). Treatment with Cytochalasin-D disrupted the filaments as evident also in the loss of structure in deformed cells.

It was shown that treatments with CytoD and LPS result in changes in the amount and in the organization of actin structure, both in the relaxed and in the deformed situations.

### Cell elastic behavior depends on cell activation

In the validation study, it was observed that the Capillary Micromechanics technique allows differentiating between not-treated monocytic cells and monocytic cells with strongly disrupted actin structure. In fact, both the compressive and the shear modulus exhibit distinct changes, based on cell treatment.

It was shown that the mechanical behavior of the monocytes also strongly depends on the activation of the cells, as can be clearly seen in the stress-strain graphs in Figures 3 and 4. For the compressive modulus shown in Fig. 3, both control and LPS-treated cells show a clear stiffening behavior with increasing deformation, which can be approximately described by using two linear regimes as shown in Fig. 2. Hence, a smaller modulus in the small deformation region (*K_1_*) and a larger modulus in the larger deformation region (*K_2_*) can be distinguished. However, for LPS-treated cells the transition to the higher modulus happens at a lower strain compared to non-treated cells. This earlier stiffening of the activated cells might be due to the polymerization of cortical actin after activation, as shown in Fig. 5. The inset in Figure 3 shows the value of the compressive modulus for the untreated and LPS activated cells in both strain regimes. The LPS-treated cells have a *K_1_* of 3,4·10^3^±647 Pa compared to 783±101 Pa of NT cells (increase of 340%) and a *K_2_* of 7·10^3^±2·10^3^ Pa compared to 4·10^3^±2,3·10^3^ Pa of NT cells (increase of 73%). The cyto-D treated cells do not show any stiffening effect for the compressive modulus and the behavior remains linear at a low modulus, a behavior that could be caused by a disruption of the main cytoskeletal supporting structure.

Figure 4 shows the results for the shear modulus. Again, the non-treated cells show clear stiffening behavior, i.e. the behavior can be divided into a shear modulus at low strains (*G_1_*), and a shear modulus at larger strains (*G_2_*). The LPS-treated cells, however, show a much less pronounced stiffening behavior for shear than for compression, and the shear modulus remains relatively small for higher strains. This behavior results in a reduction of shear modulus of LPS-treated cells compared to NT cells both at small (62±24 Pa compared to 83±36 Pa, decrease of 25%) and at high deformations (63,2±28 Pa compared to 517±161 Pa, decrease of 88%) as shown in the inset of Fig. 4, in contrast to the behavior for the compressive modulus. As for the compressive modulus, the shear modulus of the cyto-D treated cells is not divided into two regions and is low for the entire strain range observed.

In conclusion, activation of the HL60 cells with LPS results in an increase in their compressive elastic modulus but a decrease in their shear elastic modulus, especially at high deformation. This drop in the magnitude of the shear modulus might be due to disruption of cytoskeleton by increased stress or to remodeling of cytoskeletal structure as in monocyte diapedesis.

## Discussion

The quantification of mechanical properties of monocytes can give insight into the development of cardiovascular diseases, such as atherosclerosis. For this reason, the present study aimed to investigate the biophysical properties of monocytic cells with a newly developed technique based on cell deformation in a glass capillary by application of external fluid pressure. The deformation of monocytic cells in the tip of the capillary allows the measurement of the full elastic response of the cell, reducing the amount of experiments and cells required. The mechanical properties were successfully measured and they are dependent on the treatment mimicking activation. Interestingly, upon activation the cells show an increase in resistance in compression, but a lower resistance to shape change in shear. This increase in cell deformability can be physiologically correlated to cell transmigration through the endothelial layer upon activation.

Apart from monitoring the full elastic response of a single circulating cell in a microcapillary tip, the benefit of the method applied in our study is that it enables precise quantification of cell mechanical properties for a large range of deformations, relevant for scientific insights into cell deformation behavior and as input for computational models. Furthermore, its ease-of-use is higher than that of common micropipette aspiration techniques and enables probing the deformation of the entire cell, rather than just a small sub-volume. However, because the technique is rather time consuming, its application for routine diagnostic purposes seems limited. So far, a vast range of techniques has been used for the measurement of the mechanical properties of circulating cells. These include filtration techniques [8], [19]–[21], micropipette aspiration [21]–[24], and atomic force microscopy (AFM) [25]. As these techniques often probe different parts of the cell, reported values for cell stiffness and the elastic response of cells upon activation vary considerably with the type of method used. More importantly, most techniques provide distinct or momentary values, as they are not able to measure the elastic response of a single cell over a large range of deformations. Cell filtration, for instance, can provide insight into the average behavior of a cell population, but cannot measure the properties at the single cell level. Micropipette aspiration, on the other hand, can be used for single cell probing, but is generally applied to determine cell membrane cortical tension or cytoplasmic viscosity and when used for whole cell aspiration, it mainly gives momentary results. AFM is not ideal for measuring the mechanical properties of circulating, non-adherent cells because they tend to slip while pressured with the cantilever. In addition, AFM is a very local technique that probes only a small and superficial portion of the cell.

Changes in elastic and adhesive response are usually related to cellular modifications. Signalling generated by chemokines can cause a series of biochemical reactions that might result in reorganization of cytoskeletal structures. It is well known that the actin network and the actin binding proteins provide the main contribution to the mechanical properties of living cells. In fact, it was shown in previous studies on leukocytes [6], [10], [26]–[28] and it was confirmed in our analysis that the increase in stiffness induced by activation compounds is related to polymerization and reorganization of actin. To rationalize this behavior, a simplified conceptual cell model (File S1) was used to estimate the changes in the compressive and the shear modulus of the cell, respectively, upon the re-organization of actin into a shell-like structure. In the case of re-arrangement of the total amount of actin from a homogeneous network to a localized rim in the submembrane region, the model is in qualitative agreement with the experimental results; predicting a significantly different behavior for the compressive and the shear modulus. In fact, the model predicts that the compressive modulus strongly increases while the shear modulus, depending on the thickness of the submembrane region, can exhibit a slight decrease.

The low resistance to shape deformation of activated cells might also be caused by re-arrangement of other cytoskeletal proteins or of the membrane structure. The low shear modulus can also be explained from disruption of filamentous actin or of the bridging of actin cross-linking proteins. Previous studies showed that F-actin depolymerizes and softens under high deformation, especially at low concentrations of actin cross-linking protein [11], [29]. In the present study, cells were stained inside the pipette at two different ranges of deformation. However, the immunostaining of actin structure of circulating cells was not sufficient to show differences in protein organization and crosslinking. Further experiments are required to explain this abnormal behavior of activated cells (e.g. staining of other cytoskeletal component under cellular deformation).

The experiment was performed both at room temperature and at physiological temperature. At RT, it was possible to detect differences between not-treated and activated cells. As such, the technique might be useful to detect mechanical changes as a biomarker for cell activation and disease progression in clinical settings. At physiological temperature, it was shown that leukocytes subjected to compression undergo activation. In fact, mechanical deformation comparable to the range experienced in the microcirculation causes changes in cell structure and function. In previous studies on neutrophil compression, it was observed that upregulation of adhesion molecules and reorganization of cell cytoskeleton with projection of pseudopods happened mainly at physiological temperature [26], [30], [31]. We indeed see comparable behavior as the other leukocyte subpopulation. In our study, the analysis at physiological temperature confirmed this dynamic response of monocytes subjected to mechanical stress. In fact, non-treated cells, deformed in the tip of the capillary, showed projection of pseudopods, and an elastic response similar to LPS-treated cells. However, it was interesting to notice that LPS-cells at physiological temperature were more compressible than activated cells at room temperature (Fig. S4). This behavior at physiological conditions might allow a better adhesion to the vascular wall and a quicker diapedesis to the damaged tissue.

In summary, our data show that non-treated and activated cells can be distinguished on the basis of elastic response. Cells activated by LPS show a higher compressive modulus compared to not-treated cells. The resistance to shape deformation, on the other hand, is lower for LPS-treated cells, even at high deformation. These findings are important for understanding the link between changes in mechanical properties of cells and their function; as such they are also of relevance for the development of quantitative models that describe the (ab)normal behavior of monocytes in circulation and for the prediction of vascular events.

## Supporting Information

Figure S1 **Bright field images of untreated cells deformed in the microcapillary at 37°C.** The formation of pseudopods at this temperature is indicated by the red arrows. At room temperature, non-treated cells did not form pseudopods (see Fig. 5).
(TIF)

Figure S2 **Scatterplot of compressive stress versus volumetric strain for non-treated (NT, GREEN), activated LPS-treated (RED) and Cyto-D treated (BLUE) cells at 37°C.**
(TIF)

Figure S3 **Scatterplot of the characteristic differential stress (p_wall_ – p)/2 as a function of strain ε_r_ – ε_z_ for non-treated (NT, GREEN), activated LPS-treated (RED) and cytoD-treated (BLUE) cells at 37°C.**
(TIF)

Figure S4 **Scatterplot of the compressive stress as a function of the volumetric strain for activated LPS-treated cells at 21°C (BLUE) and at 37°C (RED).**
(TIF)

Video S1 **Friction analysis in a flat non-tapered 4 μm-wide glass capillary.**
(MP4)

File S1 **Simplified conceptual cell model to estimate the changes in the compressive and the shear modulus of the cell.**
(DOCX)
