# Supplementary figures and images for: Monocytic Cells Become Less Compressible but More Deformable upon Activation
Source: PLoS One. 2014 Mar 27;9(3):e92814. doi: 10.1371/journal.pone.0092814 (PMC3968036; doi:10.1371/journal.pone.0092814)

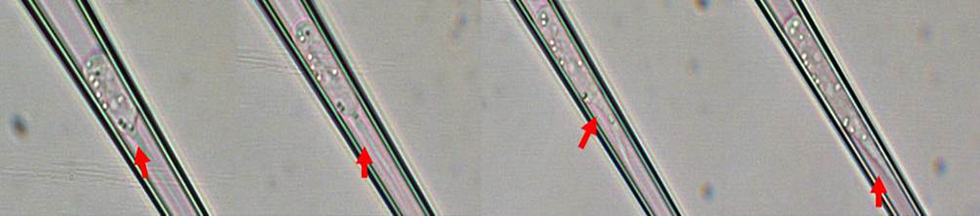

Supplement: Figure S1 — Bright field images of untreated cells deformed in the microcapillary at 37°C. The formation of pseudopods at this temperature is indicated by the red arrows. At room temperature, non-treated cells did not form pseudopods (see Fig. 5). (TIF) [file pone.0092814.s001.tif]

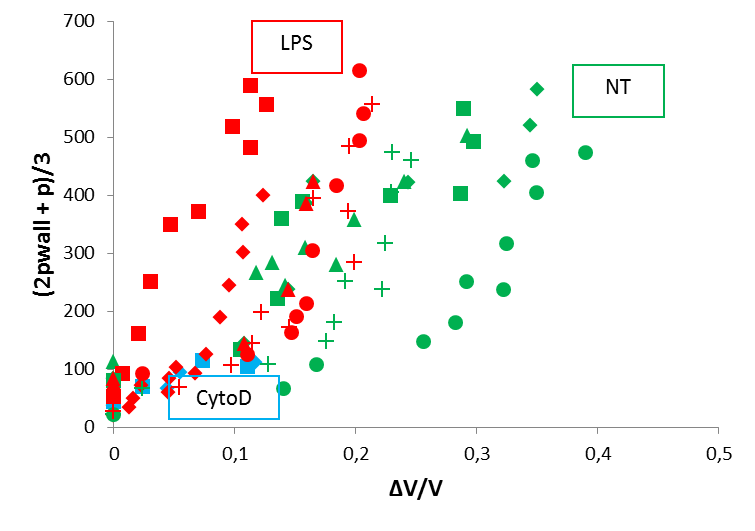

Supplement: Figure S2 — Scatterplot of compressive stress versus volumetric strain for non-treated (NT, GREEN), activated LPS-treated (RED) and Cyto-D treated (BLUE) cells at 37°C. (TIF) [file pone.0092814.s002.tif]

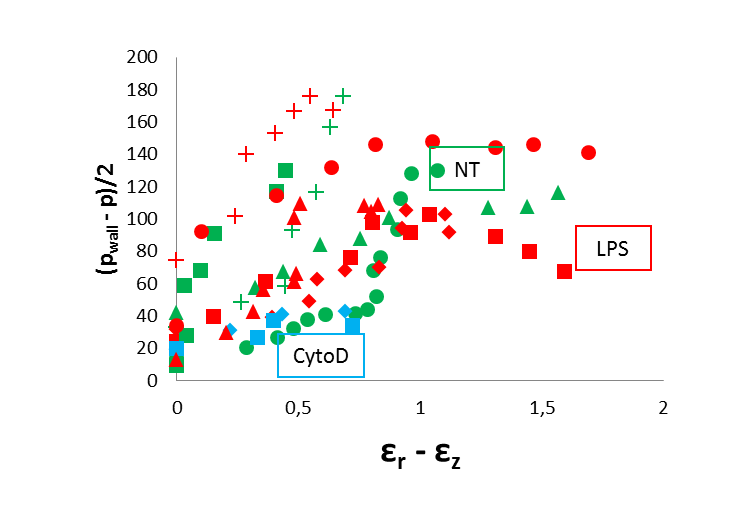

Supplement: Figure S3 — Scatterplot of the characteristic differential stress (pwall – p)/2 as a function of strain εr – εz for non-treated (NT, GREEN), activated LPS-treated (RED) and cytoD-treated (BLUE) cells at 37°C. (TIF) [file pone.0092814.s003.tif]

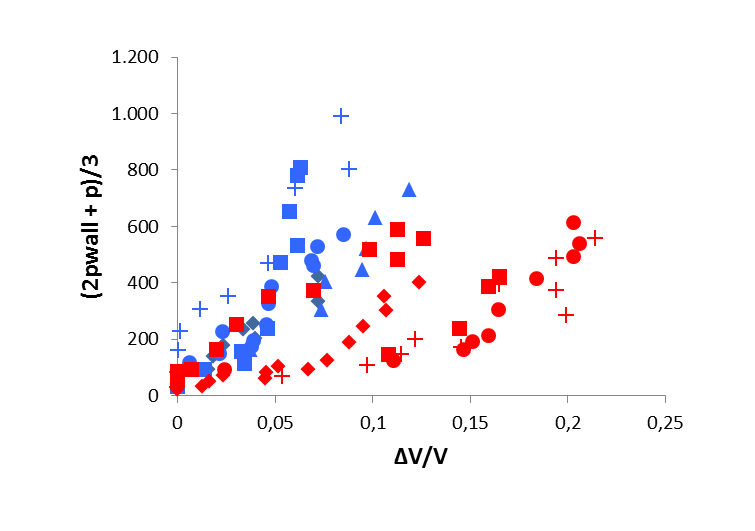

Supplement: Figure S4 — Scatterplot of the compressive stress as a function of the volumetric strain for activated LPS-treated cells at 21°C (BLUE) and at 37°C (RED). (TIF) [file pone.0092814.s004.tif]
