# Supplementary material for: Monocytic Cells Become Less Compressible but More Deformable upon Activation
Source: PLoS One. 2014 Mar 27;9(3):e92814. doi: 10.1371/journal.pone.0092814 (PMC3968036; doi:10.1371/journal.pone.0092814)
Supplement: File S1 — Simplified conceptual cell model to estimate the changes in the compressive and the shear modulus of the cell. (DOCX) [file pone.0092814.s006.docx]

Upon activation, actin re-organizes from a homogenous network to a localized rim in the submembrane region. We used a simplified conceptual model to estimate the changes in compressive and shear modulus.

*Φ_0_*

*K=K_0_, G=G_0_*

*Φ_c_*

*K_c_, G_c_*

*Φ_s_*

*K_s_, G_s_*

*R_c_*

*R*

Control

Not treated

Activated

LPS-treated

Actin

We assume that the compressive and shear elastic moduli *K* and *G* scale with the concentration of actin (or volume fraction Φ) as *K* and G ~ $\Phi^{\frac{5}{2}}$, as has been previously theoretically predicted by scaling arguments [1] and confirmed by experiment [2].

We further assume that actin reorganization occurs from an initially homogeneous concentration Φ_o_ into a core-shell structure with a spherical core of lower concentration Φ_c_ and a shell of higher concentration Φ_s_. We assume that the total amount of actin stays constant, thus

$\Phi_{c}V_{c}+\Phi_{s}V_{s}=\Phi_{0}V$, or

$$\alpha^{3}\Phi_{c}+(1-\alpha^{3})\Phi_{s}=\Phi_{0}$$

where$\alpha$is the core-to-shell size ratio, *α = R_c_/R_s_*. As a result, the volume fractions in the core and the shell are related via

$\Phi_{c}=\frac{\Phi_{0}-(1-\alpha^{3})\Phi_{s}}{\alpha^{3}}$, and

$\Phi_{s}=\frac{\Phi_{0}-\alpha^{3}\Phi_{c}}{1-\alpha^{3}}$.

The local compressive and shear moduli in the core and in the shell are then given as

$$K_{c}=\left(\frac{\Phi_{c}}{\Phi_{0}}\right)^{\frac{5}{2}}K_{0}$$

$$G_{c}=\left(\frac{\Phi_{c}}{\Phi_{0}}\right)^{\frac{5}{2}}G_{0}$$

In the case of re-arrangement of the total amount of actin from a diffuse structure to a ring structure at the cortex (Φ_s_= 0),

*Φ_c_*

*K_c_, G_c_*

*Φ_s_*

*K_s_, G_s_*

*R_c_*

*R*

Activated

LPS-treated

the volume fraction and the moduli within the shell material are given as

$$\Phi_{s}=\frac{\Phi_{0}}{1-\alpha^{3}}$$

$$K_{s}=\left(\frac{1}{1-\alpha^{3}}\right)^{\frac{5}{2}}K_{0}$$

$$G_{s}=\left(\frac{1}{1-\alpha^{3}}\right)^{\frac{5}{2}}G_{0}$$

1

α

α

Assuming that the compressive modulus is governed by a volume average of core and shell, we obtain

$$K=\alpha^{3}K_{c}+(1-\alpha^{3})\left(\frac{1}{1-\alpha^{3}}\right)^{\frac{5}{2}}K_{0}$$

for the compressive modulus of the entire core-shell structure. To estimate the corresponding shear modulus, we consider the core-shell structure as a cube-shaped structure. In this case, along any given axis, there are three separate segments in parallel, where the front and rear segments have a shear modulus of *G*_s_, whereas the middle segment consists of three separate segments in series. As a result; the shear modulus of the entire object is then given as

$G\approx\alpha\left[\frac{G_{c}G_{s}}{(1-\alpha^{3})G_{s}+\alpha^{3}G_{c}}\right]+(1-\alpha)G_{s}$,

such that finally for the case of *K_c_* and *G_c_*= 0, we obtain

$$K=\left(\frac{1-\alpha}{1-\alpha^{3}}\right)^{\frac{3}{2}}K_{0}$$

$$G=\left(\frac{1-\alpha}{(1-\alpha^{3})^{\frac{5}{2}}}\right)G_{0}$$

the model is in qualitative agreement with the experimental results, predicting a significantly different behavior for the compressive and the shear modulus. In fact, the model predicts that the compressive modulus strongly increases while the shear modulus, depending on the thickness of the submembrane region, can exhibit a slight decrease, as shown in the following graph.

Figure 1 – Plot of K (-) and G (--) as a function of the aspect ratio α. The inset shows how the ratio between the storage and loss modulus changes with aspect ratio. These graphs are referring to the case where we assume K_c_ and G_c_ = 0.

1. Morse DC (1998) Viscoelasticity of Concentrated Isotropic Solutions of Semiflexible Polymers. Macromolecules 31: 7044–7067.

2. Gardel M, Shin J, MacKintosh F, Mahadevan L, Matsudaira P, et al. (2004) Scaling of F-Actin Network Rheology to Probe Single Filament Elasticity and Dynamics. Phys Rev Lett 93: 188102. Available: http://link.aps.org/doi/10.1103/PhysRevLett.93.188102. Accessed 17 October 2013.
